# Ethyl 2-(4-methyl­benzo­yl)-2,3-dihydro-1*H*-indene-2-carboxyl­ate

**DOI:** 10.1107/S1600536813005801

**Published:** 2013-03-06

**Authors:** Jiachen Xiang, Tingting Hu, Jungang Wang

**Affiliations:** a Key Laboratory of Pesticides and Chemical Biology of the Ministry of Education, College of Chemistry, Central China Normal University, Wuhan 430079, People’s Republic of China

## Abstract

The title compound, C_20_H_20_O_3_, contains two fused rings with a quaternary carbon centre connecting *p*-toluoyl and eth­oxy­carbonyl groups. The dihedral angle between the fused benzene ring and the three-C-atom plane (derived from O=C—C—C=O) is 82.5 (4)°, whereas the dihedral angle between the planes of the benzene rings is 53.4 (2)°. In the crystal, molecules are linked *via* C—H⋯O_ester_ hydrogen bonds, forming chains propagating along [010].

## Related literature

For the preparation and crystal engineering studies of the title compound, see: Singh & Paul (2006 ▶); Wang & Wu (2012 ▶).
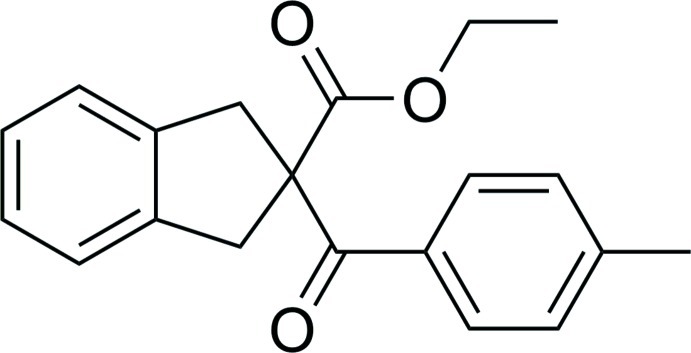


## Experimental

### 

#### Crystal data

C_20_H_20_O_3_
*M* *_r_* = 308.37
Monoclinic, 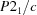

*a* = 8.1957 (14) Å
*b* = 6.1287 (10) Å
*c* = 32.995 (5) Å
β = 93.014 (3)°
*V* = 1655.0 (5) Å^3^
*Z* = 4
Mo *K*α radiation
μ = 0.08 mm^−1^
*T* = 298 K
0.12 × 0.10 × 0.10 mm

#### Data collection

Bruker APEXII CCD diffractometer
Absorption correction: multi-scan (*SADABS*; Sheldrick, 1997 ▶) *T*_min_ = 0.990, *T*_max_ = 0.992
11945 measured reflections
3241 independent reflections
2482 reflections with *I* > 2σ(*I*)
*R*_int_ = 0.025

#### Refinement

*R*[*F* ^2^ > 2σ(*F* ^2^)] = 0.049
*wR*(*F* ^2^) = 0.187
*S* = 1.09
3241 reflections
210 parameters
H-atom parameters constrained
Δρ_max_ = 0.27 e Å^−3^
Δρ_min_ = −0.25 e Å^−3^

### 

Data collection: *APEX2* (Bruker, 2004 ▶); cell refinement: *SAINT*(Bruker, 2004 ▶); data reduction: *SAINT*; program(s) used to solve structure: *SHELXS97* (Sheldrick, 2008 ▶); program(s) used to refine structure: *SHELXL97* (Sheldrick, 2008 ▶); molecular graphics: *SHELXTL* (Sheldrick, 2008 ▶); software used to prepare material for publication: *SHELXTL*.

## Supplementary Material

Crystal structure: contains datablock(s) I, global. DOI: 10.1107/S1600536813005801/gg2108sup1.cif

Structure factors: contains datablock(s) I. DOI: 10.1107/S1600536813005801/gg2108Isup2.hkl

Supplementary material file. DOI: 10.1107/S1600536813005801/gg2108Isup3.cml

Additional supplementary materials:  crystallographic information; 3D view; checkCIF report

## Figures and Tables

**Table 1:** Hydrogen-bond geometry (Å, °)

*D*—H⋯*A*	*D*—H	H⋯*A*	*D*⋯*A*	*D*—H⋯*A*
C1—H1⋯O1^i^	0.93	2.60	3.357 (3)	139

Symmetry code: (i) 
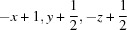
.

**Fig. 1.**
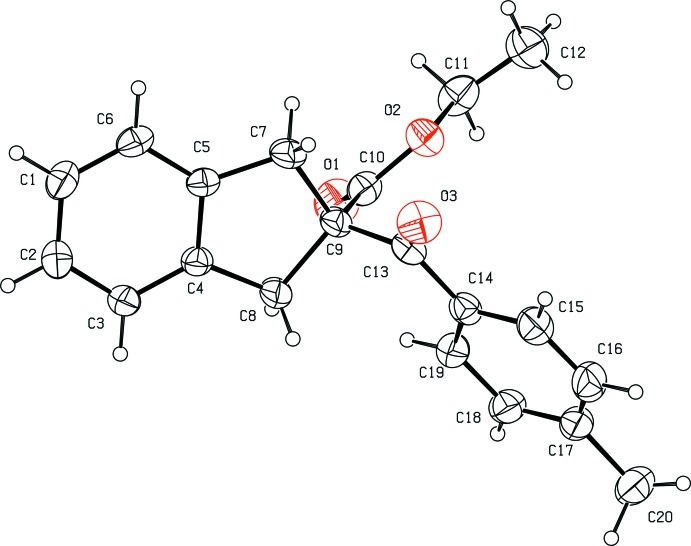
A view of (I), showing the atom-labelling scheme, with displacement ellipsoids drawn at the 30% probalility level.

## supplementary crystallographic information

### Comment

The title molecule (I)(Fig.1) was synthesized in a mixture of 1,4-dibromo-2,3- bis(bromomethyl)benzene (1.0 mmol), ethyl 3-oxo-3-(*p*-tolyl)propanoate (1.0 mmol) and Cs_2_CO_3_ (2 mmol) in DMSO (5 ml). The mixture was stirred at 40° for 30 min until almost full conversion of the substrates by TLC analysis. The resulting mixture was dropped into 100 ml 1 *M* HCl (aq) and extracted with EtOAc 3 times (3 times 50 ml). The organic extract was dried with Na_2_SO_4_, filtered and concentrated. The crude product was purified by column chromatography on silica gel (eluent: petroleum ether/EtOAc=50/1) to afford the product as a white solid.

### Experimental

The title compound was synthesized according to the reported method (Singh *et al.*; 2006 and Wang *et al.*; 2012). The ethyl 3-oxo-3-(*p*-tolyl)propanoate reacts with 1,2-bis(halomethyl)benzene to obtain the title compound *via* a two-step *C*-alkylation process. Crystals of (I) suitable for X-ray diffraction were grown by slow evaporation of a acetic ether solution of the title compound (I) at 293 K.

### Crystal data

**Table:**

C_20_H_20_O_3_	*F*(000) = 656
*M**_r_* = 308.37	*D*_x_ = 1.238 Mg m^−^^3^
Monoclinic, *P*2_1_/*c*	Mo *K*α radiation, λ = 0.71073 Å
Hall symbol: -P 2ybc	Cell parameters from 3624 reflections
*a* = 8.1957 (14) Å	θ = 2.5–24.5°
*b* = 6.1287 (10) Å	µ = 0.08 mm^−^^1^
*c* = 32.995 (5) Å	*T* = 298 K
β = 93.014 (3)°	Block, colourless
*V* = 1655.0 (5) Å^3^	0.12 × 0.10 × 0.10 mm
*Z* = 4	

### Data collection

**Table:**

Bruker APEXII CCD diffractometer	3241 independent reflections
Radiation source: fine-focus sealed tube	2482 reflections with *I* > 2σ(*I*)
Graphite monochromator	*R*_int_ = 0.025
φ and ω scans	θ_max_ = 26.0°, θ_min_ = 1.2°
Absorption correction: multi-scan (*SADABS*; Sheldrick, 1997)	*h* = −10→10
*T*_min_ = 0.990, *T*_max_ = 0.992	*k* = −7→7
11945 measured reflections	*l* = −40→36

### Refinement

**Table:**

Refinement on *F*^2^	Primary atom site location: structure-invariant direct methods
Least-squares matrix: full	Secondary atom site location: difference Fourier map
*R*[*F*^2^ > 2σ(*F*^2^)] = 0.049	Hydrogen site location: inferred from neighbouring sites
*wR*(*F*^2^) = 0.187	H-atom parameters constrained
*S* = 1.09	*w* = 1/[σ^2^(*F*_o_^2^) + (0.1176*P*)^2^ + 0.1336*P*] where *P* = (*F*_o_^2^ + 2*F*_c_^2^)/3
3241 reflections	(Δ/σ)_max_ = 0.008
210 parameters	Δρ_max_ = 0.27 e Å^−^^3^
0 restraints	Δρ_min_ = −0.25 e Å^−^^3^

### Special details

**Table:**

Geometry. All e.s.d.'s (except the e.s.d. in the dihedral angle between two l.s. planes) are estimated using the full covariance matrix. The cell e.s.d.'s are taken into account individually in the estimation of e.s.d.'s in distances, angles and torsion angles; correlations between e.s.d.'s in cell parameters are only used when they are defined by crystal symmetry. An approximate (isotropic) treatment of cell e.s.d.'s is used for estimating e.s.d.'s involving l.s. planes.
Refinement. Refinement of *F*^2^ against ALL reflections. The weighted *R*-factor *wR* and goodness of fit *S* are based on *F*^2^, conventional *R*-factors *R* are based on *F*, with *F* set to zero for negative *F*^2^. The threshold expression of *F*^2^ > σ(*F*^2^) is used only for calculating *R*-factors(gt) *etc*. and is not relevant to the choice of reflections for refinement. *R*-factors based on *F*^2^ are statistically about twice as large as those based on *F*, and *R*- factors based on ALL data will be even larger.

### Fractional atomic coordinates and isotropic or equivalent isotropic displacement parameters (Å^2^)

**Table:**

	*x*	*y*	*z*	*U*_iso_*/*U*_eq_	
C1	0.7866 (3)	0.2221 (4)	0.25704 (6)	0.0666 (6)	
H1	0.8421	0.2668	0.2809	0.080*	
C2	0.8114 (3)	0.0156 (4)	0.24231 (7)	0.0693 (6)	
H2	0.8860	−0.0765	0.2559	0.083*	
C3	0.7266 (2)	−0.0567 (3)	0.20748 (6)	0.0555 (5)	
H3	0.7424	−0.1972	0.1978	0.067*	
C4	0.6182 (2)	0.0832 (3)	0.18736 (5)	0.0463 (4)	
C5	0.5970 (2)	0.2939 (3)	0.20139 (5)	0.0452 (4)	
C6	0.6805 (2)	0.3627 (3)	0.23682 (6)	0.0552 (5)	
H6	0.6647	0.5027	0.2467	0.066*	
C7	0.4828 (3)	0.4205 (3)	0.17361 (7)	0.0626 (6)	
H7A	0.5406	0.5355	0.1602	0.075*	
H7B	0.3963	0.4854	0.1885	0.075*	
C8	0.5092 (3)	0.0359 (3)	0.15049 (7)	0.0655 (6)	
H8A	0.4358	−0.0840	0.1556	0.079*	
H8B	0.5732	−0.0015	0.1276	0.079*	
C9	0.4114 (2)	0.2508 (3)	0.14203 (6)	0.0514 (5)	
C10	0.2314 (3)	0.2265 (3)	0.14927 (6)	0.0555 (5)	
C11	−0.0233 (3)	0.4180 (4)	0.14107 (7)	0.0726 (7)	
H11A	−0.0434	0.4237	0.1698	0.087*	
H11B	−0.0798	0.2922	0.1293	0.087*	
C12	−0.0820 (3)	0.6211 (5)	0.12057 (9)	0.0863 (8)	
H12A	−0.0303	0.7449	0.1336	0.129*	
H12B	−0.1983	0.6324	0.1222	0.129*	
H12C	−0.0554	0.6173	0.0926	0.129*	
C13	0.4316 (2)	0.3270 (3)	0.09827 (6)	0.0516 (5)	
C14	0.3593 (2)	0.1945 (3)	0.06402 (6)	0.0513 (5)	
C15	0.3657 (3)	0.2784 (4)	0.02492 (7)	0.0676 (6)	
H15	0.4123	0.4147	0.0212	0.081*	
C16	0.3042 (3)	0.1633 (5)	−0.00819 (7)	0.0791 (7)	
H16	0.3093	0.2236	−0.0340	0.095*	
C17	0.2350 (3)	−0.0398 (5)	−0.00395 (7)	0.0705 (7)	
C18	0.2282 (3)	−0.1239 (4)	0.03492 (7)	0.0665 (6)	
H18	0.1819	−0.2607	0.0385	0.080*	
C19	0.2881 (2)	−0.0102 (3)	0.06837 (6)	0.0578 (5)	
H19	0.2811	−0.0703	0.0941	0.069*	
C20	0.1698 (4)	−0.1689 (6)	−0.04011 (8)	0.1051 (11)	
H20A	0.2398	−0.2916	−0.0442	0.158*	
H20B	0.1664	−0.0777	−0.0638	0.158*	
H20C	0.0616	−0.2196	−0.0354	0.158*	
O1	0.1677 (3)	0.0787 (3)	0.16597 (5)	0.0896 (6)	
O2	0.15098 (16)	0.4028 (2)	0.13532 (4)	0.0625 (4)	
O3	0.5094 (2)	0.4910 (3)	0.09153 (5)	0.0775 (5)	

### Atomic displacement parameters (Å^2^)

**Table:**

	*U*^11^	*U*^22^	*U*^33^	*U*^12^	*U*^13^	*U*^23^
C1	0.0753 (13)	0.0748 (15)	0.0488 (11)	−0.0053 (11)	−0.0071 (9)	−0.0038 (10)
C2	0.0733 (13)	0.0787 (15)	0.0548 (12)	0.0152 (12)	−0.0078 (10)	0.0051 (11)
C3	0.0662 (12)	0.0488 (11)	0.0516 (11)	0.0107 (9)	0.0038 (9)	0.0000 (8)
C4	0.0530 (9)	0.0411 (9)	0.0452 (9)	0.0012 (7)	0.0056 (7)	−0.0007 (7)
C5	0.0496 (9)	0.0402 (9)	0.0465 (10)	−0.0029 (7)	0.0097 (7)	−0.0031 (7)
C6	0.0675 (11)	0.0487 (11)	0.0502 (11)	−0.0103 (9)	0.0109 (9)	−0.0092 (8)
C7	0.0749 (13)	0.0380 (10)	0.0738 (14)	0.0074 (9)	−0.0077 (10)	−0.0111 (9)
C8	0.0928 (15)	0.0363 (10)	0.0649 (13)	0.0133 (9)	−0.0206 (11)	−0.0072 (9)
C9	0.0586 (10)	0.0358 (9)	0.0589 (11)	0.0047 (8)	−0.0059 (8)	−0.0017 (8)
C10	0.0712 (12)	0.0477 (11)	0.0480 (10)	−0.0093 (9)	0.0066 (9)	0.0021 (8)
C11	0.0517 (11)	0.1032 (19)	0.0637 (13)	−0.0118 (11)	0.0122 (9)	−0.0123 (13)
C12	0.0489 (12)	0.108 (2)	0.1017 (19)	0.0111 (12)	0.0005 (11)	−0.0099 (16)
C13	0.0441 (9)	0.0453 (10)	0.0659 (12)	0.0038 (8)	0.0074 (8)	0.0053 (9)
C14	0.0455 (9)	0.0539 (11)	0.0550 (11)	0.0080 (8)	0.0078 (7)	0.0051 (8)
C15	0.0675 (12)	0.0726 (14)	0.0641 (13)	0.0099 (11)	0.0151 (10)	0.0132 (11)
C16	0.0814 (15)	0.104 (2)	0.0525 (13)	0.0238 (15)	0.0096 (11)	0.0089 (13)
C17	0.0571 (11)	0.0984 (18)	0.0555 (13)	0.0277 (12)	−0.0020 (9)	−0.0150 (12)
C18	0.0634 (12)	0.0707 (14)	0.0649 (13)	0.0048 (10)	−0.0021 (9)	−0.0147 (11)
C19	0.0625 (11)	0.0587 (12)	0.0521 (11)	0.0014 (9)	0.0013 (8)	0.0008 (9)
C20	0.097 (2)	0.146 (3)	0.0700 (16)	0.0411 (19)	−0.0142 (14)	−0.0384 (17)
O1	0.1113 (14)	0.0733 (11)	0.0858 (13)	−0.0225 (10)	0.0211 (10)	0.0235 (9)
O2	0.0509 (8)	0.0652 (9)	0.0725 (9)	0.0009 (6)	0.0132 (6)	0.0094 (7)
O3	0.0783 (10)	0.0644 (9)	0.0909 (12)	−0.0230 (8)	0.0139 (9)	0.0072 (8)

### Geometric parameters (Å, º)

**Table:**

C1—C6	1.372 (3)	C11—C12	1.484 (4)
C1—C2	1.374 (3)	C11—H11A	0.9700
C1—H1	0.9300	C11—H11B	0.9700
C2—C3	1.384 (3)	C12—H12A	0.9600
C2—H2	0.9300	C12—H12B	0.9600
C3—C4	1.379 (3)	C12—H12C	0.9600
C3—H3	0.9300	C13—O3	1.216 (2)
C4—C5	1.385 (2)	C13—C14	1.489 (3)
C4—C8	1.499 (3)	C14—C15	1.392 (3)
C5—C6	1.389 (3)	C14—C19	1.395 (3)
C5—C7	1.493 (3)	C15—C16	1.374 (4)
C6—H6	0.9300	C15—H15	0.9300
C7—C9	1.564 (3)	C16—C17	1.378 (4)
C7—H7A	0.9700	C16—H16	0.9300
C7—H7B	0.9700	C17—C18	1.386 (3)
C8—C9	1.559 (3)	C17—C20	1.506 (3)
C8—H8A	0.9700	C18—C19	1.374 (3)
C8—H8B	0.9700	C18—H18	0.9300
C9—C10	1.514 (3)	C19—H19	0.9300
C9—C13	1.535 (3)	C20—H20A	0.9600
C10—O1	1.195 (2)	C20—H20B	0.9600
C10—O2	1.335 (3)	C20—H20C	0.9600
C11—O2	1.454 (2)		
			
C6—C1—C2	120.55 (19)	O2—C11—H11A	110.3
C6—C1—H1	119.7	C12—C11—H11A	110.3
C2—C1—H1	119.7	O2—C11—H11B	110.3
C1—C2—C3	120.7 (2)	C12—C11—H11B	110.3
C1—C2—H2	119.6	H11A—C11—H11B	108.6
C3—C2—H2	119.6	C11—C12—H12A	109.5
C4—C3—C2	118.89 (19)	C11—C12—H12B	109.5
C4—C3—H3	120.6	H12A—C12—H12B	109.5
C2—C3—H3	120.6	C11—C12—H12C	109.5
C3—C4—C5	120.47 (17)	H12A—C12—H12C	109.5
C3—C4—C8	127.69 (17)	H12B—C12—H12C	109.5
C5—C4—C8	111.81 (16)	O3—C13—C14	120.20 (19)
C4—C5—C6	119.98 (18)	O3—C13—C9	120.44 (18)
C4—C5—C7	111.40 (16)	C14—C13—C9	119.33 (16)
C6—C5—C7	128.61 (17)	C15—C14—C19	117.6 (2)
C1—C6—C5	119.33 (19)	C15—C14—C13	117.97 (19)
C1—C6—H6	120.3	C19—C14—C13	124.38 (17)
C5—C6—H6	120.3	C16—C15—C14	121.1 (2)
C5—C7—C9	105.30 (15)	C16—C15—H15	119.4
C5—C7—H7A	110.7	C14—C15—H15	119.4
C9—C7—H7A	110.7	C15—C16—C17	121.3 (2)
C5—C7—H7B	110.7	C15—C16—H16	119.4
C9—C7—H7B	110.7	C17—C16—H16	119.4
H7A—C7—H7B	108.8	C16—C17—C18	117.8 (2)
C4—C8—C9	105.09 (15)	C16—C17—C20	121.6 (3)
C4—C8—H8A	110.7	C18—C17—C20	120.5 (3)
C9—C8—H8A	110.7	C19—C18—C17	121.6 (2)
C4—C8—H8B	110.7	C19—C18—H18	119.2
C9—C8—H8B	110.7	C17—C18—H18	119.2
H8A—C8—H8B	108.8	C18—C19—C14	120.5 (2)
C10—C9—C13	109.40 (15)	C18—C19—H19	119.8
C10—C9—C8	112.61 (16)	C14—C19—H19	119.8
C13—C9—C8	110.23 (16)	C17—C20—H20A	109.5
C10—C9—C7	107.08 (16)	C17—C20—H20B	109.5
C13—C9—C7	111.68 (16)	H20A—C20—H20B	109.5
C8—C9—C7	105.78 (15)	C17—C20—H20C	109.5
O1—C10—O2	123.5 (2)	H20A—C20—H20C	109.5
O1—C10—C9	126.9 (2)	H20B—C20—H20C	109.5
O2—C10—C9	109.51 (15)	C10—O2—C11	118.56 (16)
O2—C11—C12	106.95 (18)		
			
C6—C1—C2—C3	−1.9 (3)	C8—C9—C10—O2	−169.98 (16)
C1—C2—C3—C4	1.0 (3)	C7—C9—C10—O2	74.16 (19)
C2—C3—C4—C5	1.2 (3)	C10—C9—C13—O3	124.95 (19)
C2—C3—C4—C8	−177.0 (2)	C8—C9—C13—O3	−110.7 (2)
C3—C4—C5—C6	−2.5 (3)	C7—C9—C13—O3	6.6 (2)
C8—C4—C5—C6	176.01 (18)	C10—C9—C13—C14	−56.9 (2)
C3—C4—C5—C7	176.28 (18)	C8—C9—C13—C14	67.4 (2)
C8—C4—C5—C7	−5.2 (2)	C7—C9—C13—C14	−175.30 (15)
C2—C1—C6—C5	0.6 (3)	O3—C13—C14—C15	−8.0 (3)
C4—C5—C6—C1	1.6 (3)	C9—C13—C14—C15	173.84 (16)
C7—C5—C6—C1	−176.9 (2)	O3—C13—C14—C19	170.76 (19)
C4—C5—C7—C9	7.9 (2)	C9—C13—C14—C19	−7.4 (3)
C6—C5—C7—C9	−173.47 (17)	C19—C14—C15—C16	−0.1 (3)
C3—C4—C8—C9	178.54 (18)	C13—C14—C15—C16	178.77 (18)
C5—C4—C8—C9	0.2 (2)	C14—C15—C16—C17	−0.4 (3)
C4—C8—C9—C10	−112.09 (18)	C15—C16—C17—C18	0.5 (3)
C4—C8—C9—C13	125.43 (18)	C15—C16—C17—C20	−179.0 (2)
C4—C8—C9—C7	4.5 (2)	C16—C17—C18—C19	0.0 (3)
C5—C7—C9—C10	112.94 (18)	C20—C17—C18—C19	179.4 (2)
C5—C7—C9—C13	−127.31 (17)	C17—C18—C19—C14	−0.5 (3)
C5—C7—C9—C8	−7.4 (2)	C15—C14—C19—C18	0.6 (3)
C13—C9—C10—O1	135.3 (2)	C13—C14—C19—C18	−178.23 (18)
C8—C9—C10—O1	12.4 (3)	O1—C10—O2—C11	0.7 (3)
C7—C9—C10—O1	−103.5 (2)	C9—C10—O2—C11	−176.98 (16)
C13—C9—C10—O2	−47.0 (2)	C12—C11—O2—C10	−176.51 (19)

### Hydrogen-bond geometry (Å, º)

**Table:**

*D*—H···*A*	*D*—H	H···*A*	*D*···*A*	*D*—H···*A*
C7—H7*A*···O3	0.97	2.28	2.762 (3)	110
C8—H8*A*···O1	0.97	2.45	2.883 (3)	107
C1—H1···O1^i^	0.93	2.60	3.357 (3)	139

Symmetry code: (i) −*x*+1, *y*+1/2, −*z*+1/2.
